# Complex regional pain syndrome in a living kidney donor

**DOI:** 10.1590/1806-9282.20251778

**Published:** 2026-07-10

**Authors:** Tuğba Aydın, Fatma Nur Kesiktaş, Dilara Ekici Zincirci

**Affiliations:** 1 Istanbul Physical Medicine and Rehabilitation Training and Research Hospital – Istanbul, Turkey.; 2 Prof. Dr. Cemil Taşcıoğlu City Hospital, Department of Physical Medicine and Rehabilitation – Istanbul, Turkey.

Dear Editor,

Complex regional pain syndrome (CRPS) is characterized by disproportionate pain in relation to the triggering event, as well as sensory, autonomic, trophic, and motor abnormalities^1^. Common triggers include fractures, sprains, blunt trauma, and surgical procedures^2^. However, reports of CRPS in the context of transplantation have almost exclusively focused on recipients and have most often been linked to immunosuppressive therapy^2,3,4,5^. To our knowledge, there are no previously published case reports describing the development of CRPS in a living kidney donor. In this letter, we aim to draw attention to the need to broaden the differential diagnosis of post-donation limb pain by presenting a case of CRPS that developed in the weeks following donor nephrectomy.

Approximately 6 weeks after donating her left kidney to her cousin, a 42-year-old woman presented with pain in the left ankle and foot. She rated the pain as 8/10 on a visual analog scale (VAS) during activity and 6/10 at rest and described it as burning and disproportionate to any minor strain or trauma. She also reported increased sensitivity to touch and movement of the affected limb. Physical examination revealed edema, erythema, increased warmth, and marked hyperalgesia involving the ankle and metatarsal areas. Soft tissue swelling was additionally confirmed on plain radiography. Circumferential measurements demonstrated a 2-cm increase in diameter 10 cm below the knee, a 3-cm increase around the ankle malleolus, and a 2-cm increase around the metatarsus in the left lower extremity compared with the contralateral side. Active movements of the ankle were slightly limited because of pain, but there was no objective motor deficit or trophic change in the skin, hair, or nails. Cranial nerve examination, motor strength, reflexes, sensation, and cerebellar testing were otherwise normal.

Laboratory tests were as follows: C-reactive protein 4.1 mg/L (<5 mg/L), erythrocyte sedimentation rate 20 mm/h (0–20 mm/h), blood urea nitrogen 30 mg/dL (17–43 mg/dL), creatinine 1.07 mg/dL (0.51–0.95 mg/dL), estimated glomerular filtration rate (Cockcroft–Gault formula) 69.9 mL/min/1.73 m^2^ (120–130 mL/min/1.73 m^2^), serum albumin 4.5 g/dL (3.5–5 g/dL), and alkaline phosphatase 199 U/L (30–120 U/L). A urine dipstick test showed no proteinuria, and the spot urine protein/creatinine ratio was 56 mg/g (0–150 mg/g). Normal acute-phase reactants and the absence of systemic findings made inflammatory disease unlikely. There was no history of trauma, and fracture was excluded on the basis of normal plain radiography.

Lumbar magnetic resonance imaging (MRI) revealed no evidence of disc herniation or nerve root compression, and there were no signs of radiculopathy. An electromyography study performed two weeks earlier at another clinic, because of transient numbness in the foot, was normal. Arterial and venous Doppler ultrasonography of the left lower limb, performed 1 week earlier due to swelling and color changes, showed normal flow without evidence of deep vein thrombosis or significant peripheral arterial disease. Taken together, these findings effectively excluded radiculopathy/entrapment neuropathy (lumbar MRI and electroneuromyography), vascular pathology (arterial and venous Doppler ultrasound), an acute-phase response suggestive of systemic inflammation, and generalized edema due to renal causes; nephrotic-range proteinuria was absent, and the swelling was unilateral.

A three-phase bone scintigraphy was then performed. The perfusion and blood-pool phases demonstrated increased tracer uptake in the calcaneus and metatarsal bones of the left foot compared with the right side, and the delayed (metabolic) phase showed mildly increased osteoblastic activity in the same regions. These findings were compatible with, though not specific for, early CRPS. Clinically, the patient had disproportionate limb pain, reported symptoms in at least three Budapest categories (sensory: burning pain and mechanical hypersensitivity; vasomotor: color and temperature changes; sudomotor/edema: unilateral swelling), and showed signs in at least two categories on examination (sensory: hyperalgesia; vasomotor: erythema and increased warmth; sudomotor/edema: measurable edema). Accordingly, the patient fulfilled the Budapest clinical criteria for CRPS^6,7,8,9,10^. The findings on three-phase bone scintigraphy and the mild elevation of alkaline phosphatase were supportive of this diagnosis, correlating with the observed clinical picture.

The rehabilitation program, conducted 5 days per week for 3 weeks, consisted of contrast bath therapy, followed by stretching and isometric strengthening exercises for the ankle. Contrast bath therapy was selected to support desensitization of the affected limb and to help improve vasomotor regulation.

For contrast baths, two containers were used: one containing cold water at 10–15°C and the other containing warm water at 40–42°C. The affected foot was alternately immersed in cold and warm water. Each session consisted of five 1-min cold immersions and five 3-min warm immersions, following standard protocols used in CRPS rehabilitation. The patient tolerated the treatment well.

Medical treatment was initiated with pregabalin 150 mg/day (75 mg twice daily). After 1 week, the dose was titrated to 300 mg/day (150 mg twice daily), which was maintained thereafter, and was well-tolerated. At the 2-month follow-up, the patient rated her pain as 3/10 on the VAS during activity and 0/10 at rest. Her walking difficulties had markedly decreased, and her performance of daily life activities had improved. On physical examination, there was no longer any difference in circumference 10 cm below the knee, around the ankle malleolus, or around the metatarsus between the left and right lower extremities.

In the literature, cases of CRPS after transplantation have been reported almost exclusively in recipients and are mostly attributed to immunosuppressive agents, particularly calcineurin inhibitors and, less commonly, mechanistic target of rapamycin inhibitors^4,5^. The donor in this case had no exposure to immunosuppressive therapy; therefore, this observation suggests that surgical trauma and perioperative stressors alone may be sufficient to trigger CRPS in susceptible individuals. Although donor nephrectomy is anatomically remote from the affected limb, CRPS arising after non-orthopedic and anatomically distant trunk or abdominal procedures has been described, including exploratory laparotomy, appendectomy, and laparoscopic umbilical port-site surgery^2,10^. These reports support the concept that, in at least a subset of patients, CRPS is driven predominantly by central sensitization and dysregulation of sympathetic efferent–afferent coupling rather than direct nerve injury in the symptomatic region^1,7,10^. Perioperative factors such as patient positioning, transient immobilization, and systemic inflammatory responses may further contribute to the initiation of CRPS in predisposed patients^1,2^. Early diagnosis and rehabilitation-centered management are crucial to shorten the disease course, avoid unnecessary investigations and medication changes, and improve functional outcomes^6,7^.

Consequently, post-transplant CRPS should not be regarded as a complication confined to recipients. In the follow-up of living kidney donors, CRPS should be considered in the differential diagnosis when disproportionate limb pain is accompanied by autonomic and trophic changes, and early rehabilitation should be prioritized.

## Data Availability

The datasets generated and/or analyzed during the current study are available from the corresponding author upon reasonable request.

## References

[1] Ferraro MC, O’Connell NE, Sommer C, Goebel A, Bultitude JH, Cashin AG (2024). Complex regional pain syndrome: advances in epidemiology, pathophysiology, diagnosis, and treatment.. Lancet Neurol.

[2] Moretti A, Gimigliano F, Paoletta M, Bertone M, Liguori S, Toro G (2021). Complex regional pain syndrome type I following non-orthopedic surgery: case report and narrative review.. Diagnostics.

[3] Tillmann FP, Jäger M, Blondin D, Oels M, Rump LC, Grabensee B (2008). Post-transplant distal limb syndrome: clinical diagnosis and long-term outcome in 37 renal transplant recipients.. Transpl Int.

[4] Vanacker A, Vandewiele I, Aerts P, Groof J, Maes B (2007). Reflex sympathetic dystrophy in a renal transplant patient treated with sirolimus.. Nephrol Dial Transplant.

[5] Ybarra J, Crespo M, Torregrosa JV, Fuster D, Campistol JM, Oppenheimer F (2003). Reflex sympathetic dystrophy syndrome in renal transplanted patients under immunosuppression with tacrolimus.. Transplant Proc.

[6] Harnik MA, Kesselring P, Ott A, Urman RD, Luedi MM (2023). Complex regional pain syndrome (CRPS) and the value of early detection.. Curr Pain Headache Rep.

[7] Melf-Marzi A, Böhringer B, Wiehle M, Hausteiner-Wiehle C (2022). Modern principles of diagnosis and treatment in complex regional pain syndrome.. Dtsch Arztebl Int.

[8] Perez RS, Collins S, Marinus J, Zuurmond WW, Lange JJ (2007). Diagnostic criteria for CRPS I: differences between patient profiles using three different diagnostic sets.. Eur J Pain.

[9] Harden RN, Bruehl S, Perez RS, Birklein F, Marinus J, Maihofner C (2010). Validation of proposed diagnostic criteria (the “Budapest Criteria ”) for complex regional pain syndrome.. Pain.

[10] Jokonya L, Mungazi S, Mduluza-Jokonya TL, Kalangu KKN (2021). Truncal complex regional pain syndrome, myth or reality: case report.. Int J Surg Case Rep.

